# Making chiral molecular knots and links stereospecifically

**DOI:** 10.1093/nsr/nwad041

**Published:** 2023-02-16

**Authors:** Huang Wu, Chun Tang, Long Zhang, J Fraser Stoddart

**Affiliations:** Department of Chemistry, Northwestern University, USA; Department of Chemistry, Northwestern University, USA; Department of Chemistry, Northwestern University, USA; Department of Chemistry, Northwestern University, USA; School of Chemistry, University of New South Wales, Australia; Stoddart Institute of Molecular Science, Department of Chemistry, Zhejiang University, China; ZJU-Hangzhou Global Scientific and Technological Innovation Center, China

**Figure 1.**
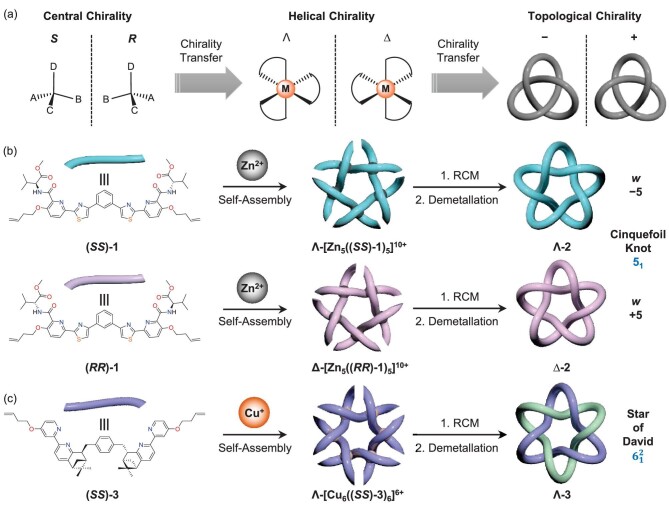
(a) Hierarchical chirality transfer from stereogenic centers to helical metal centers, and eventually to topologically chiral structures. (b) The synthesis of an enantiomeric pair of knots from pentameric circular helicates obtained by the self-assembly of the chiral strands (*SS*)-1/(*RR*)-1 with Zn^2+^ ions. (c) The synthesis of the homochiral Star of David [2]catenane from a hexameric circular helicate obtained by the self-assembly of a chiral strand (*SS*)-3 with Cu^+^ ions. RCM, ring-closing olefin metathesis. Adapted from [3,4].

Topological structures [1,2], i.e., knots and links, grace the macroscopic world in the form of biomorphs and artificial tools, all the way down to the molecular scale, such as catenated DNA and phytochromes. Chirality inherently exists in structures in which mirror-image topologies cannot be superimposed upon themselves without disassembling the topological structures. Although the chirality we are most familiar with is based on stereogenic centers, topological chirality can arise in molecules even in the absence of such centers. The stereospecific synthesis of a molecule with specific topological handedness requires controlling the entropically demanding pathways associated with knotting and linking. From a practical point of view, the stereospecific syntheses of molecules with topological structures can be controlled by the presence of stereogenic centers. Understanding how the chirality of stereogenic centers can be transferred to topological structures (Fig. 1a) is a matter of fundamental importance.

Recently, Zhang *et al.* [3,4] at East China Normal University have proposed and realized the selective syntheses of an enantiomeric pair of cinquefoil (5_1_) knots and a homochiral Star of David ($6_1^2$) [2]catenane as a result of hierarchical chirality transfer from stereogenic centers to both the knots and the link. The authors demonstrated (Fig. 1b) that the absolute configurations of the d/l-valine residues within the ligands, i.e., (*SS*)-1 and (*RR*)-1, determine the helical chirality of the circular helicates Λ-[Zn_5_((*SS*)-1)_5_](OTf)_10_ and Δ-[Zn_5_((*RR*)-1)_5_](OTf)_10_. After treatment of the two metallic circular helicates with the Hoveyda-Grubbs catalyst to connect the terminal olefins (Fig. 1b), two tight metallic cinquefoil knots, i.e., Λ-[Zn_5_2](OTf)_10_ and Δ-[Zn_5_2](OTf)_10_, were obtained enantioselectively in the impressively high yield of ∼90%. Demetallation of both knots with Li_2_S affords the wholly organic enantiomeric cinquefoil knots, i.e., Λ-2 and Δ-2, in ∼30% yields. The syntheses of single-handed knots indicate that the exploitation of stereogenic centers in ligand strands to control the helical chiralities of circular helicates, and dictate the handedness of the corresponding knots, is highly effective.

Zhang *et al.* [4] have also employed their chirality-transfer strategy in the stereospecific synthesis of a Star of David [2]catenane (Fig. 1c). They employed a chiral bipyridine ligand (*SS*)-3, which was first developed by von Zelewsky and co-workers [5] in the 1990s, to construct a topologically chiral $6_1^2\ $link. They built up the catenated structure in a stepwise fashion (Fig. 1c) by employing the chiral ligand (*SS*)-3 to afford the hexameric copper(I) circular helicate Λ-[Cu_6_((*SS*)-3)_6_](PF_6_)_6_ and converted it into the single-handed star-shaped link by ring-closing olefin metathesis (RCM). The Cu^+^ ions can be removed by treatment with the chelating agent ethylenediaminetetraacetate to afford the wholly organic homochiral Star of David [2]catenane Λ-3.

These breakthroughs are timely and certain to arouse the interest of chemists investigating topological chirality and developing related properties. With the increase in understanding of chirality transfer from stereogenic centers to topologically chiral structures, we can look forward to witnessing the enantioselective syntheses of more complex topologically chiral structures and to developing their applications in chiral separations, information transmissions, asymmetric catalysis, and more into the bargain.
